# A Systematic Review of Lemierre’s Syndrome With a Focus on Ophthalmologic Complications

**DOI:** 10.7759/cureus.9326

**Published:** 2020-07-21

**Authors:** Suhas P Dasari, Pinky Jha

**Affiliations:** 1 Internal Medicine, Medical College of Wisconsin, Wauwatosa, USA

**Keywords:** lemierre's syndrome, necrobacillosis, fusobacterium, ophthalmologic, occuloplastic, abducens, occular, septic cavernous sinus thrombosis, diplopia

## Abstract

Lemierre’s syndrome (LS), once known as “the forgotten disease,” is a rare and potentially life-threatening condition that has had a gain in incidence over the last 30 years due to a variety of factors that could include changes in antibody prescription patterns, particularly in regard to the treatment of pharyngitis/tonsillitis. Due to its low incidence and broad spectrum of symptoms, LS does not have an obvious clinical diagnosis and can confuse the clinician managing the patient. Furthermore, it is equally difficult to treat patients suffering from LS as it requires a multidisciplinary approach from multiple subspecialties. Thus, communication between hospitalists, radiologists, otolaryngologists, neurologists, and ophthalmologists is critical towards quickly diagnosing the disease condition so that prompt antibiotics, anticoagulation, and surgical intervention can occur. Atypical presentations can also exist, making the diagnosis and management exponentially more challenging. Ophthalmologic symptoms are a particularly rare and atypical presentation of LS. These rare symptoms in LS can be terrifying for patients and providers alike; yet, there does not seem to be any modern medical literature that summarizes ophthalmologic complications for LS patients. To our knowledge, this is the first systematic review of LS with a focus on ophthalmologic complications that has been done. The main objective of this review paper is to provide an up-to-date literature review of LS epidemiology, pathophysiology, diagnosis, and treatment while also performing a novel systematic review of reported cases of LS with ophthalmological complications. We hope to bring more awareness towards LS and its atypical presentations so that physicians will be better able to rapidly diagnose and treat their patients in order to minimize long-term morbidity and mortality.

## Introduction and background

Lemierre’s syndrome (LS), also known as human necrobacillosis, is a rare and potentially life-threatening condition that is characterized by internal jugular vein thrombosis and septic embolism following an oropharyngeal infection. It is most commonly caused by *Fusobacterium necrophorum* (*F. necrophorum*). *F. necrophorum* is an obligate anaerobic gram-negative bacillus that is a commensal organism of the oropharynx. Infection caused by *F. necrophorum* was first identified in 1900 and was described in detail by French physician Andre Lemierre in 1936 following a cohort of 20 cases with post-anginal septicemia [1-2]. In 1980, Vogel and Horger described the classic triad associated with LS which included *F. necrophorum* bacteremia that led to internal jugular vein thrombosis [3]. According to Olson et al., the modern diagnostic criteria include a history of “anginal illness of the oropharynx within the preceding four weeks, evidence of metastatic lesions in the lungs or another remote site, and evidence of internal jugular vein thrombophlebitis or isolation of *F. necrophorum* from the blood or another sterile site” [1, 4-5]. Further complicating the clinical treatment of LS is the presence of atypical symptoms, such as the rare cases of LS with ophthalmological involvement. Due to their low incidence, cases with ocular complications of LS can easily confuse a clinician, especially when a multidisciplinary approach is not used. Moreover, these symptoms can be particularly stress evoking for the patient and their family. Despite the morbidity associated with ophthalmological manifestations of LS, there seems to be a paucity of medical literature that addresses this presentation of the disease. In order to address this need, we performed the first systematic review of LS with a focus on ophthalmologic complications that has been done, to our knowledge.

## Review

Epidemiology

LS is a disease of young, previously healthy adults with an average age of 19 to 22 years old [1]. In Lemierre’s 1936 publication, he outlined the initial description of LS in 20 cases, 18 of whom died, during the pre-antibiotic era [2]. With its initial presentation, there was a very high mortality rate until antibiotics became prevalent in clinical practice. The widespread introduction of antibiotics for streptococcal pharyngitis in the subsequent years greatly reduced the incidence with no published cases in the 1950s or 1960s [6]. This led to LS to gain the name “the forgotten disease.” However, there has been a recent rise in reported/published cases of LS in the decades that followed with studies showing an incidence of 0.8 - 1.5 million per year worldwide between 1970 to 2007 [4, 7].

Karkos et al. performed a systematic review in 2009 with a MEDLINE® search using the terms LS, post-anginal septicemia, *Fusobacterium*, and internal jugular vein thrombosis, in which they found an upwards trend of published cases of LS in the post-antibiotic world with six cases in the 1980s, 50 cases in the 1990s, and 121 cases from 2001 - 2008 [6]. They specifically analyzed 84 of these cases (114 patients) that had *Fusobacterium* culture-confirmed cases of LS with radiographic imaging showing thrombophlebitis of the internal jugular vein [6]. In their review, they found an average age of 22 with equal prevalence in men and women; they also found a low mortality of 5%, a high morbidity of 52% (defined as a prolonged hospital or intensive care unit (ICU) course), and a median hospital stay of 25 days [6]. The most common sources of infection in their analysis was tonsillitis (37%), pharyngitis (30%), and involvement of the chest/lower respiratory tract (25%) [6]. The most common presenting symptom was a sore throat with a chest x-ray showing consolidation in 75% of cases, though 10% of cases had normal radiographs [6]. In an impressive review by Riordan in 2007, 393 cases were analyzed between 1970 and 2007 by identifying patients with necrobacillosis or suspected systemic infection with *F. necrophorum* originating in the head and neck; of these 393 cases, 222 cases met the modern criteria for LS that was previously defined in this review article [1]. For patients with necrobacillosis and throat infection, 89% presented as culture-confirmed cases with *F. necrophorum*, the most common pathogen associated with LS [1, 6]. Riordan reported an average age of 19 years old with a male predominance of close to a 2:1 ratio [1]. Numerous data points suggest that the incidence of LS rose during the 1980s - 1990s with culture-positive *F. necrophorum* LS showing a sustained increase in incidence between 1995 - 2004. A more recent systematic review from 2009 - 2014 by Blessing et al. showed a mortality of 2% in LS and that cases of LS that presented initially with oropharyngeal involvement had longer hospital courses and higher morbidity [7].

There are several proposed theories as to why there has been a recent uptick in literature-reported cases of LS. This could potentially be due to increases in antibiotic resistance, changes in prescription patterns (especially with the increase in awareness of antibiotic resistance), or a trend in literature/publishing. Karkos et al. also proposed that the trend towards the use of weekly antibiotics (like a cephalosporin) over daily antibiotics led to reduced coverage of the *Fusobacterium* species in the population of young adults with sore throats, tonsillitis, or other oropharyngeal infections [6]. Riordan also postulated that the guidelines that drive antibiotic treatment of suspected group A streptococcal pharyngitis may play a role in this trend, but the incidence of LS is currently too low to alter antibiotic prescription guidelines [1]. Taken together, all of these trends in modern medicine allow for a greater proportion of simple oropharyngeal illnesses to transform into *Fusobacterium* septicemias and increase the risk of developing LS.

Microbiology

*F. necrophorum* is the most common pathogen responsible for causing LS, and any discussion regarding the diagnosis, management, and treatment of LS begins with understanding this organism. The *Fusobacterium* species is a group of Gram-negative obligate anaerobic bacilli, and *F. necrophorum* is the most virulent species of *Fusobacterium* [8]. It is the most clinically relevant bacterium in any discussion of LS. Though *F. necrophorum* is the most common cause of LS, it is important to note that Gram-positive cocci have caused LS as well [5, 9].

Virulence factors of *F. necrophorum* include adhesins, endotoxins, hemolysins, and leukotoxins, which contribute to necrotic abscess formation [1]. Hemagglutinin is especially pathogenic and greatly increases *F. necrophorum* morbidity within the context of LS by aggregating platelets, leading to thrombophlebitis, septic emboli, thrombocytopenia, and even disseminated intravascular coagulation [1, 6]. Additionally, there is a synergistic role with other facultative bacteria that use up oxygen and lower the local reduction potential to create a protective anaerobic environment for *F. necrophorum*, specifically, protecting it from superoxide dismutase. In return, leukotoxins from *F. necrophorum* protect these oxidizing bacteria from phagocytosis [1].

While multiple sources state that *F. necrophorum* is a part of the normal flora and is made pathogenic during infection of the oropharynx [4, 8], others would argue that *F. necrophorum* must be externally acquired. Riordan specifically argued that there was no convincing culture evidence to show that *F. necrophorum* was part of the oropharyngeal flora with only one molecular study, using reverse transcription-polymerase chain reaction (RT-PCR), showing that this pathogen could be part of the normal flora of a healthy individual [1]. Despite this, *F. necrophorum* was shown to have an increased prevalence in patients with a sore throat; this was confirmed by polymerase chain reaction (PCR) [1]. Specifically, chronic tonsillitis was associated with a change in microbial flora and generally presented with more anaerobes and an increased prevalence of foul breath with the *Fusobacterium* species being one of the most commonly isolated species in these situations [1]. It is still unclear if *F. necrophorum* has a causative role in recurrent tonsillitis or whether it is just simply present in increased numbers in patients with recurrent tonsillitis [1]. Regardless, it was clear that tonsils with increased fibrosis and signs of chronic inflammation into the adjacent tissues had increased the growth of *F. necrophorum* with enhanced pathogenicity [1]. Additionally, there was some evidence to suggest an association between infectious mononucleosis and *F. necrophorum,* though there was no strong evidence to support a mechanism of action [1, 8]. It is also possible that the association is simply due to coinfection and an increased prevalence in the young adult patient population [1, 8].

Pathophysiology

LS is a medical emergency that is typically driven by an anaerobic oropharyngeal bacterial infection, leading to thrombophlebitis of the internal jugular vein and metastatic spread to distant organs via septic emboli. The Gram-negative anaerobic bacillus *F. necrophorum* is the usual culprit of LS in young, previously healthy, adults. The classic triad of presenting symptoms in most patients with LS includes pharyngotonsillitis, internal jugular venous thrombosis, and metastatic abscess [8]. While thrombophlebitis of the internal jugular vein is a classic feature of LS, it is not required to be present for a formal diagnosis of the condition. In fact, there are many reports of LS with positive cultures for *F. necrophorum,* despite having no imaging showing thrombosis in the veins of the head and neck. Thus, the modern diagnostic criteria of LS are characterized by a recent history of anginal illness within the preceding four weeks, metastatic spread of septic emboli to distant organs, and either evidence of internal jugular vein thrombophlebitis or isolation of F. necrophorum from blood or another sterile site [1, 4-5]. Riordan would argue that all patients with LS had *Fusobacterium* septicemia, and those with culture-negative LS simply had a *Fusobacterium *species infection that was not detected [1]. While this may be true, this has not been accepted by the medical community as of yet, and clinical diagnosis of LS does not require a *Fusobacterium* culture to be present so long as there is radiological evidence of internal jugular vein thrombophlebitis.

Generally speaking, LS begins with a harmless oropharyngeal infection that leads to more systemic consequences as pathogenic bacteria invade into the local vasculature. Pathogenesis occurs from the entry of the causal organism through the oropharyngeal mucosa from trauma, inflammation, or tissue destruction [8]. After passing through the mucosal barrier, post-anginal sepsis can develop as the organism directly spreads from an abscess through the deeper loose connective cervical tissues, spreads via hematogenous mechanisms, or spreads via a lymphogenic mechanism in order to extend into the veins of the head or neck with the most frequent route being from the tonsil into the ipsilateral internal jugular vein [1, 9-10]. Regardless of which path the bacteria takes and which vein it is able to seed within, the pathogenic organism will eventually activate platelets, activate the coagulation cascade, and cause inflammation leading to thrombus formation and LS disease progression. The spread of septic emboli from the internal jugular vein, or whichever vein the bacterium has seeded, can lead to the involvement of distant organs within the body.

Infection with *F. necrophorum* exists on a spectrum of disease. Necrobacillosis is any human infection with *F. necrophorum *regardless of symptoms [1]. Post-anginal sepsis is the complication of thrombophlebitis after a case of tonsillitis, which generally presented with septicemia and metastatic lesions in published cases. LS is complex in description with some authors wanting it to be only for post-anginal cases (i.e., from tonsillitis) and others suggesting that infection of any site in the oropharynx is adequate to call it LS. Some authors also argue about thrombophlebitis of the internal jugular vein (IJV) being a defining requirement, whereas others require septic emboli, especially to the lung, as markers for LS. Lemierre’s original paper described a septic illness with metastatic spread and these metastatic lesions were the defining feature required to classify a case as LS [2]. Because antibiotics can make blood cultures appear sterile (up to 33% of patients can receive antibiotics before admission), Riordan argued that there should be no requirement for bacterial culture in order to diagnose LS so long as there was the presence of metastatic lesions [1, 11]. Furthermore, there has been an extensive amount of cases of classical LS with metastatic lesions and culture-confirmed presence of *F. necrophorum* without any imaging evidence of internal jugular vein thrombophlebitis with ultrasound, contrast-enhanced computed tomography (CECT), or magnetic resonance imaging (MRI) modalities [1]. Taken together, with the concept that only an infection arising from the oropharynx should be included in the classification of LS, we can create modern diagnostic criteria that have been used to define LS in the literature over the past 10 - 15 years [11]. 

Based on these criteria, Riordan found 222 cases of LS between 1970 and 2007 with 89% being positive for *F. necrophorum* on blood culture and 48% positive for internal jugular vein thrombosis [1]. Sinus thrombosis occurs in about 25% of cases. The majority of patients will be young, acutely ill, and have unstable vitals. They will initially present with a prolonged sore throat and fever that has often progressed to cause swelling of the throat/neck, respiratory symptoms, and septic symptoms [5]. Labs will show leukocytosis with a neutrophil predominance and elevated C-reactive protein (CRP); additionally, there can be signs of organ failure based on where metastatic septic emboli spread to. Imaging would show thrombosis in the veins of the head and neck (often the internal jugular vein) and metastatic spread to distant organs due to septic emboli which will lead to abscess formation [5]. The clinical features of LS depend upon which end organs are involved with metastatic spread. Septic emboli typically lead to the involvement of the pulmonary capillaries, and pulmonary lesions are the most common finding in up to 92% patients [1, 10, 12]. The second most common manifestation of metastasis would be septic arthritis that presents in 13% - 27% of patients and classically affects the hip, knee, or shoulder joint [10]. Upon arthrocentesis, septic arthritis in LS will present as foul, malodorous pus in the margins of the joint capsule [1]. Other complications include skin/soft tissue infections, intra-abdominal sepsis, and endocarditis/pericarditis [1].

Systematic review of ophthalmologic complications

LS can also present with uncommon symptoms and ocular involvement is a particularly rare manifestation of LS [7, 9, 13]. Based on our comprehensive literature review, we could only identify 27 total patients between 2009 and 2019 that met our criteria for LS and who presented with any symptom involving the orbit, the extraocular muscles, the optic nerve, the oculomotor nerve, the trochlear nerve, the abducens nerve, or the cavernous sinus. This is outlined in Table 1. For reference, Johannesen et al. identified 137 LS patients using very similar inclusion criteria for LS over just five years (2010 - 2015) [5].

**Table 1:** Comprehensive Literature Review of Cases of Lemierre’s Syndrome With Ophthalmological Complications Over the Past 10 Years (2009 - 2019) Articles were found using PubMed search terms Lemierre, Fusobacterium, or necrobacillosis combined with ophthalmologic, ocular, eye, diplopia, exophthalmos, cavernous, or ophthalmoplegia. All case reports found were read and any relevant cases cited within the report were cross-referenced, analyzed, and compiled into our table. Cases that did not meet the three requirements that define LS outlined in our systematic review were not included. CN: cranial nerve; CSF: cerebrospinal fluid; CST: cavernous sinus thrombosis; EBV: Epstein-Barr virus; F: female; GDC: Guglielmi detachable coil; ICA: internal carotid artery; IJV: internal jugular vein; IOP: interocular pressure; IV: intravenous; L: left; M: male; MCA: middle cerebral artery; MRSA: methicillin-resistant S. aureus; MSSA: methicillin-sensitive S. aureus; OD: oculus dexter; OS: oculus sinister; OU: oculus uterque; PD: pupillary distance; R: right; SOV: superior ophthalmic vein; TMP-SMX: trimethoprim-sulfamethoxazole; URI: upper respiratory infection

Author	Patient (Age/Sex)	Source of Infection	Ophthalmic Involvement	Sites of Embolism, Abscess, and Vascular Disease	Causal Organism	Antibiotics, Anticoagulation, and Surgical Intervention	Outcome
Hegde et al. 2009 [21]	23 F	L parapharyngeal abscess	R proptosis (26 mm OD vs 22 mm OS), diplopia, decreased visual acuity (6/24 OD and 6/5 OS), chemosis, and painful limitation of gaze	R CST	Positive blood culture of F. necrophorum	IV benzylpenicillin with metronidazole; no anticoagulation; tonsillectomy	Full recovery
Lee et al. 2009 [22]	3 M	Sore throat	Trochlear nerve palsy leading to R head tilt and strabismus	R IJV, L IJV, and L external jugular vein thrombosis	S. viridans and S. salivarius detected on blood culture	IV ceftriaxone and clindamycin; unspecified anticoagulation; 12 mm L inferior oblique surgical recession	At 6 months, the patient remained orthotropic with primary gaze and L gaze with 2 prism L hypertropia in R gaze
Aouad et al. 2010 [20]	4 M	R peritonsillar abscess	Photophobia and R periorbital edema	R IJV thrombosis extending into sigmoid and superior petrosal sinuses, multiple orbital abscesses, CST, multiple brain/lung abscesses, and endocarditis of mitral valve	MSSA was cultured in blood, CSF, and pus from an abscess	Unspecified broad-spectrum antibiotics; unspecified anticoagulation; drainage of peritonsillar abscess	Patient died on Day 26
Chacko et al. 2010 [23]	19 M	Sore throat (positive serology EBV)	None	L SOV thrombosis, frontal lobe empyema, and bibasilar septic emboli of lungs	Positive blood culture of F. necrophorum	Unspecified antibiotic regimen; no anticoagulation; endoscopic sinus surgery and frontal craniotomy	Not stated
Lim et al. 2010 [16]	32 M	Sore throat	Diplopia with reduced visual acuity (20/50 OS, 20/22 OD), bilateral total ophthalmoplegia, ptosis, mydriasis, and absence of pupillary light reflex bilaterally	R IJV thrombosis, bilateral CST, bilateral ICA aneurysms, septic emboli to lungs	MRSA was isolated on blood culture	Imipenem and metronidazole; heparin; endoscopic sphenoidotomy and GDC coiling with four-vessel angiography	At 2 years, visual acuity and ophthalmoplegia improved with some limitation in R eye upward gaze and some remaining photophobia
Peer Mohammed and Carr 2010 [24]	14 M	Sore throat with recent L ear infection	R abducens nerve palsy	Leptomeningitis with thrombosis of L sigmoid sinus and IJV	Positive blood culture of F. necrophorum	Ceftriaxone and acyclovir, then ceftriaxone and metronidazole	At 3 months, the patient had full recovery of CNVI
Bababeygy et al. 2011 [25]	3 M	Tonsillitis and otomastoiditis	Esotropia with L abducens nerve palsy	L IJV thrombosis, CST, SOV thrombosis, and sigmoid sinus thrombosis	Positive blood culture of F. Necrophorum	IV metronidazole and cefepime, then IV ceftriaxone and metronidazole; warfarin; ear tubes placed	At 5 weeks, the patient had some improvement in abducens nerve palsy
Kahn et al. 2011 [19]	45 M	Sore throat with a history of drug abuse	Worsening visual acuity (20/70 OD and 20/40 OS), R inferior chemosis, decreased extraocular motility in all directions, proptosis, and elevated IOP (34 mmHg OD 14 mmHg OS)	Bilateral SOV thrombosis, L IJV thrombosis, L intracranial dural venous sinus thrombosis, bilateral CST, bilateral orbital abscesses, and pulmonary septic emboli	S. milleri detected on blood culture	IV vancomycin, meropenem, rifampin, penicillin, and metronidazole; heparin; incision and drainage of R orbital abscess	Patient died
Kraus and Culican 2012 [26]	19 F	Monospot positive with fever and headache	L orbital pain, proptosis, ptosis, bilateral abducens nerve palsy, and blurred vision	R CST, L hypothalamic ischemic infarct, L masticator space abscess, IJV thrombosis	Blood cultures positive for F. necrophorum, A. haemolyticum, and Group C beta-hemolytic strep	Unspecified broad-spectrum antibiotics; unspecified anticoagulation; surgical sinus exploration and drainage of abscess	The patient had mild remaining ptosis with abduction defect
Krishna, et al. 2012 [27]	35 M	Not stated	Eye pain and diplopia	Thrombosis of the lateral and sigmoid sinus, as well as bilateral IJVs	Not stated	Unspecified antibiotics; unspecified anticoagulation	Not stated
Miller et al. 2012 [10]	35 M	Sore throat with positive Monospot	Binocular diplopia, binocular periorbital, R ptosis, chemosis, reduced visual acuity (20/30 OD 20/25 OS), increased intraocular pressures (24 mmHg OD, 22 mmHg OS) and limited extraocular motility in all directions	R transverse/sigmoid sinus thrombosis, R IJV thrombosis, bilateral CST, R SOV thrombosis, and septic emboli to lungs	Causal organism never identified on culture	IV vancomycin and meropenem; heparin	Patient had full recovery of visual acuity (20/20 OU) with mild remaining R abducens nerve palsy
Shibuya et al. 2012 [28]	33 M	Unclear	R exophthalmos and diplopia secondary to impaired lateral (abducens nerve) and inferior extraocular motility	L IJV thrombosis, bilateral CST, and multiple lung abscesses	Unclear	Unspecified antibiotic regimen	Unclear
Aggarwal et al. 2013 [29]	15 F	Vestibulitis of nose	L abducens nerve palsy	L IJV thrombosis and CST	Negative blood cultures	Amoxicillin-clavulanate and metronidazole, then meropenem, levofloxacin, and teicoplanin; low molecular weight heparin; right-sided empyema drained	At 6 months, the patient had a full recovery
Gutzeit et al. 2013 [12]	22 F	L tonsillitis	R abducens nerve palsy (became bilateral) with partial oculomotor palsy, mydriasis, and R exophthalmos	Bilateral CST, R SOV thrombosis, R temporal lobe abscess, L tonsil abscess, R IJV thrombosis, multiple septic emboli to lungs, and aneurysm of R ICA	Positive blood culture of F. Necrophorum	Amoxicillin-clavulanate and clindamycin, then ceftriaxone and metronidazole; heparin; surgical drainage of L tonsil abscess	Patient had full recovery of oculomotor nerve and minor R abducens palsy
Morelli et al. 2013 [30]	54 F	Retropharyngeal abscess	Ptosis and chemosis of L eye	CST, L IJV thrombosis, multiple pulmonary abscesses, retropharyngeal abscess, epidural empyema	Not stated	Not stated	Not stated
Rudski et al. 2013 [31]	21 M	One-week history of URI	Diplopia, anisocoria, and R abducens nerve palsy	R maxillary and sphenoid sinusitis, R CST, and R ICA stenosis	Sinus pus culture positive for S. viridans; mucosal biopsy positive for F. necrophorum	Oral amoxicillin-clavulanate, then IV ceftriaxone, vancomycin, and metronidazole, then amphotericin B; enoxaparin; endoscopic sinus surgery	Not stated
Stauffer et al. 2013 [17]	18 M	Retropharyngeal abscess and osteomyelitis after trauma	Progressive visual loss, L periorbital edema (became bilateral), and impaired extraocular motility	R IJV, bilateral SOV thrombosis, cerebral venous thrombosis of R sigmoid and transverse sinus	MRSA was isolated on blood culture	Ciprofloxacin and azithromycin, then clindamycin, vancomycin, piperacillin-tazobactam, and acyclovir, then vancomycin, metronidazole, rifampin, and gentamycin	Patient did not regain function of CNII with no detection of light or pupillary response to light
Olson et al. 2014 [4]	18 F	Pharyngitis with positive EBV serology	Horizontal diplopia, reduced ocular motility (OD supraduction and abduction), and reduced visual acuity (20/30 OD)	Subperiosteal abscess in R orbit, R parotid gland abscess, thrombosis of R facial vein, bilateral CST, bilateral arteritis and thrombosis of ICA, and infarcts along R MCA	Positive blood culture of F. necrophorum	Oral erythromycin, then TMP-SMX and clindamycin, then IV vancomycin, meropenem, and clindamycin (TMP-SMX added back on), then IV vancomycin and meropenem (discharge) (transitioned to oral clindamycin); enoxaparin and aspirin, then dalteparin and aspirin (discharge) (transitioned to warfarin); fine-needle aspiration of parotid gland abscess and superolateral orbitotomy of R orbital abscess. Also given 5 units botulinum toxin injection to R medial rectus muscle	At 4 weeks, the patient regained full extraocular motility and diplopia had resolved
Golan et al. 2014 [18]	41 M	Pharyngitis with R peritonsillar abscess	R-sided ophthalmoplegia with loss of pupillary light response	R IJV thrombosis, R frontoparietal subdural empyema with orbital abscess, and R fusiform aneurysm of ICA	Positive blood culture of F. necrophorum	Ceftriaxone, clindamycin, ciprofloxacin, and metronidazole; no anticoagulation; endoscopic drainage of sphenoid sinus, emergent R frontoparietal craniotomy, and drainage of intraorbital abscess	Patient discharged after finishing rehabilitation; unclear final outcome
Nishida et al. 2015 [32]	54 F	Untreated periodontal disease	Bilateral periorbital pain with associated blepharoptosis, chemosis, and disturbed eye movement	Bilateral CST, SOV thrombosis, and IJV thrombosis	Intraoral resident flora was cultured on blood (not specified)	Unspecified antibiotic; unspecified anticoagulation; treatment of periodontal disease	Full recovery
Takahashi et al. 2015 [33]	73 M	Unclear	Diplopia and R-sided ptosis (became bilateral)	Clivus osteomyelitis, steroid-responsive mass lesion in cavernous sinus, bilateral septic emboli of lungs, and L IJV thrombosis	Unclear	Flomoxef, then clindamycin, then meropenem, then ampicillin-sulbactam	Patient was discharged with full recovery and no ophthalmic symptoms
Ballester et al. 2016 [34]	59 M	“Infected mouth” with poor dental hygiene	Diplopia, ptosis, and proptosis of L eye	R pterygomaxillary abscess, L IJV thrombosis, L SOV thrombosis, and septic emboli to the lungs	Cultured G. morbillorum from abscess	IV piperacillin-tazobactam and linezolid; no anticoagulation; intraoral drainage of abscess and treatment of involved teeth	Patient had fully recovered by the time of discharge
Hama et al. 2016 [35]	39 M	Untreated periodontal disease and dental caries	R orbital pain, bilateral eyelid swelling, bilateral exophthalmos, and ophthalmoplegia	R IJV thrombosis, R CST, R sigmoid sinus thrombosis, L SOV thrombosis, and multiple lung nodules	Positive blood culture of F. necrophorum (F. nucleatum was also detected later)	Levofloxacin, then IV meropenem; no anticoagulation; treatment of periodontal disease and dental caries	Patient achieved full recovery with no neurologic deficits
Budhram et al. 2017 [36]	51 M	History of tooth extraction (4 months prior)	R partial ophthalmoplegia and ptosis (became bilateral)	L sigmoid sinus thrombosis, bilateral CST, bilateral lung nodules, and focal pachymeningitis	Blood cultures grew Actinomyces meyeri, Parvimonas micra, and Fusobacterium species	Meropenem and metronidazole; rivaroxaban; removal of the residual tooth root	Not stated
Kobayashi and Matsui 2017 [37]	46 M	R peritonsillar abscess	R palsy of oculomotor nerve, trochlear nerve, and abducens nerve	R IJV thrombosis that involved sigmoid sinus and cavernous sinus with pulmonary septic emboli	Blood cultures grew Prevotella intermedia	Unspecified antibiotic regimen; heparin; surgical drainage of peritonsillar abscess	Patient did not fully recover and had residual neurological impairment
Martel 2018 [38]	86 M	None reported	L acute orbital syndrome: visual acuity (20/40), inferior chemosis, superior orbital fissure syndrome (forehead hypoesthesia and diffuse ophthalmoplegia)	L SOV thrombosis, L CST, L IJV thrombosis, L transversal sinus thrombosis	Blood culture showed S. Intermedius and S. warneri	Amoxicillin-clavulanate; unspecified anticoagulation	There was “quick regression of redness, visual loss, and ophthalmoplegia” leading to patient discharge in 3 weeks
Vu et al. 2019 [9]	65 F	Recent sinus surgery	Bilateral abducens nerve palsies with vertical and horizontal nystagmus	R IJV thrombosis and R CST	Blood culture grew S. intermedius	Ceftriaxone, ampicillin, vancomycin, ertapenem, piperacillin-tazobactam; rivaroxaban; sinus debridement surgery	At 6 months, the final deviation was esophoria at 2 PD distance and orthophoria on near gaze

Ocular involvement occurs from septic emboli undergoing retrograde transportation in the veins of the head and neck. The spread of septic emboli from the IJV can lead to the involvement of the sinuses, causing ophthalmologic complications, meningitis, and/or cerebral abscess [1]. One of the earliest cases reporting ophthalmological involvement in LS was a case in 1912 which described a patient with tonsillitis who had developed bilateral loss of vision secondary to vitreous hemorrhage, which was later explained by cavernous sinus thrombosis that had spread from the internal jugular vein [1]. To understand the scope of ophthalmologic complications in LS, we performed a literature search which is outlined in Table 1. Recently, Vu et al. performed a literature search in 2019 where they could only identify four cases of LS where diplopia was reported [9]. Our subsequent literature search showed nine reported cases of LS where diplopia was specifically used to describe a patient symptom (Table 1). Based on our review, it is evident that while ophthalmological complications are rare in LS, they tend to present with the same pattern of symptoms, including proptosis and orbitopathy, as well as reduced visual acuity and impaired extraocular motility [10]. Additionally, while possible, bilateral ocular involvement is considered exceptionally rare and was not specifically quantified in this review [13].

In LS, when individual cranial nerves are affected, isolated palsies of the abducens nerve and trochlear nerve are most commonly involved [10]. When ocular motility is affected, the most common defect is due to an abducens nerve palsy [9]. Taken together, this would imply that the abducens nerve is relatively more susceptible to the retrograde spread of septic emboli in LS relative to the other neurovascular structures of the orbit and eye. To understand how septic emboli could cause diplopia and ophthalmological symptoms, it is important to understand the route that the abducens nerve takes from the pontomedullary junction to the lateral rectus muscle and to understand the vascular supply of the cavernous sinus. The abducens nerve runs lateral to the sphenoid bone and then pierces the dura before traversing between the dura and apex of the petrous bone [4]. Here, it enters the cavernous sinus, where it freely interacts with the venous blood within the sinus, travels along the internal carotid artery, and pierces through the orbit into the lateral rectus muscle [4, 14]. The cavernous sinuses are formed by the meningeal and periosteal layers of the dura mater and are separated from the sphenoid sinuses by a thin bone [14]. They receive blood from the superior ophthalmic veins, the cranial veins, and the sphenoparietal sinus; these extensive vascular connections make the cavernous sinus vulnerable to septic thrombosis produced by infections involving the sinuses, oropharynx, and ears [14]. In LS, this allows the retrograde spread of septic emboli from the oropharynx and the veins of the head and neck, such as the IJV, to cause cavernous sinus thrombosis and ocular symptoms, such as abducens nerve palsies, diplopia, and even reduced visual acuity or impaired pupillary response to light. As a result, septic thrombosis of the cavernous sinuses is a potentially lethal medical emergency that requires early recognition and treatment to minimize serious morbidity and mortality.

In general, cavernous sinus thrombosis presents with headaches, fever, and ocular symptoms which can include proptosis, chemosis, or cranial nerve palsies of the oculomotor nerve, trochlear nerve, and abducens nerve [15]. Additionally, impairment of CSF flow from a sinus thrombosis can lead to intracranial hypertension, papilledema, and diplopia from abducens nerve palsies, providing two mechanisms where cavernous sinus thrombosis can impair the abducens nerve [15]. Moreover, cavernous sinus involvement could lead to paralysis of the lateral rectus muscle from impingement or muscle inflammation beyond the impairment of the abducens nerve itself [4]. Thus, clinicians should strongly consider LS in the context of recent oropharyngeal infection and a new onset abducens nerve palsy [9].

Due to the morbidity associated with cavernous sinus thrombosis in LS and the lack of any comprehensive literature review on LS with ophthalmologic complications, we performed a systematic literature review to fill this void in the medical literature. This review is outlined in Table 1. Between 2009 - 2019, there were 27 cases of LS that met the criteria outlined in this paper and where the patients presented with any symptoms involving the orbit, the extraocular muscles, the optic nerve, the oculomotor nerve, the trochlear nerve, the abducens nerve, or the cavernous sinus. The subsequent data was analyzed and presented in Table 2. The most common presenting symptom was impaired extraocular motility, which included diplopia, cranial nerve (CN) III/IV/VI palsies, ophthalmoplegia, and strabismus. This occurred in 24 cases (88.89%) of the 27 patients that met the inclusion criteria; an abducens nerve palsy specifically occurred in 12 cases (44.44%). Cavernous sinus thrombosis was present in 19 cases (70.37%). The next most common presenting symptoms were blepharoptosis/ptosis (nine cases; 33.33%) and impaired visual acuity (eight cases; 29.63%). Three patients had severely impaired visual acuity on presentation (defined in Table 2 as complete loss of visual acuity/light perception or loss of pupillary light reflex). One patient had methicillin-resistant S. aureus (MRSA) bacteremia and was discharged with some recovery of visual acuity [16]. The second patient also had MRSA bacteremia and did not regain function of the optic nerve so there was no return of the pupillary light reflex [17]. The third patient had *F. necrophorum* bacteremia and had an unclear outcome, but the authors stated the patient had “improved clinically” after surgical intervention [18]. Eight cases (29.63%) made a complete recovery as defined by the authors of the respective papers, while two cases (7.41%) proved to be fatal. Both fatal cases had Gram-positive cocci bacteremia identified by blood culture [19-20]. Nine cases (33.33%) had blood culture-confirmed *F. necrophorum* and 22 cases (81.48%) had imaging that confirmed IJV thrombophlebitis. Nine cases (33.33%) had Gram-positive cocci identified on blood culture and these cases tended to have poorer outcomes. Finally, in terms of treatment, 17 cases (62.96%) received anticoagulation, 19 cases (70.37%) received some type of surgical intervention, and 14 cases (51.85%) received both.

**Table 2:** Analysis of Comprehensive Literature Review of Lemierre’s Syndrome (LS) Cases With Ophthalmological Disease Over the Past 10 Years (2009 - 2019) CN: cranial nerve; IJV: internal jugular vein

Analysis of Comprehensive Literature Review of LS Cases With Ophthalmological Disease Over a 10-Year Period (2009 - 2019)	
Total number of cases	27
Cavernous sinus thrombosis	19
Extraocular muscle dysfunction (diplopia, CN III/IV/VI palsy, ophthalmoplegia, or strabismus)	24
Abducens nerve palsy (specifically)	12
Proptosis	5
Blepharoptosis/ptosis	9
Decreased visual acuity	8
Complete loss of visual acuity/light perception or loss of pupillary light reflex	3
Elevated Intraocular pressure	2
Blood culture-confirmed F. necrophorum	9
Blood culture-confirmed Gram-positive cocci	9
Imaging-confirmed IJV thrombosis	22
Treated with anticoagulation	17
Treated with surgical intervention (includes needle aspiration, drainage of abscesses, and dental procedures)	19
Treated with both anticoagulation and surgical Intervention	14
Complete recovery (defined by respective authors)	8
Remaining gaze limitation, diplopia, or strabismus	8
Permanently impaired visual acuity (Including blindness)	1
Fatal Cases	2

Diagnosis

Early diagnosis of LS is critical as any delay in treatment can lead to a doubling in mortality from 5% to about 10% [5, 7]. Specifically, delays in treatment by four or more days have been shown to significantly worsen clinical outcomes [7]. Unfortunately, the diagnosis of LS is difficult due to its low incidence and lack of pathognomonic symptoms. Furthermore, the symptoms (and management) present over such a wide range of specialties and make it even more complicated to identify early on. As a result, clinicians are often unable to consider LS on their differential diagnosis until complications from metastatic spread occur or *F. necrophorum* is identified on culture, both of which are dangerously late in the patient’s clinical course. A combination of clinical symptoms, inflammatory markers, imaging findings, and blood cultures can be used to help clinicians identify LS as quickly as possible to minimize long-term morbidity and mortality in their patients.

Generally, the earliest clinical symptom is a nonspecific sore throat. This nonspecific sore throat may resolve by the time post-anginal symptoms begin to present but unilateral swelling of the jaw or along the sternocleidomastoid muscles (SCM), indicating thrombosis of IJV, can be an early “red flag” presenting symptom in 26% - 45% of cases before significant metastatic spread has occurred. A high level of CRP should also play a crucial role in quickly determining LS [1]. Infectious mononucleosis is frequently a primary diagnosis that is made instead of LS, but focusing on classical findings of LS, particularly unilateral swelling associated with IJV thrombosis, elevated CRP, and later, septic emboli, should guide clinical suspicion. The general pattern to classically look for includes a sore throat with a foul odor characteristic of anaerobes, followed by high fever, an elevated CRP, and eventual jugular vein thrombophlebitis with metastatic lesions [1].

An early collection of blood culture samples, ideally before antibiotic treatment, is crucial towards establishing a clear diagnosis of LS. *F. necrophorum* is notoriously difficult to culture and has a long incubation time as well, making it easy to miss [5]. This can be worrisome as septic embolization can occur while clinicians wait for culture confirmation of this slow-growing organism. Paradoxically, when initial cultures are negative, this may actually make *Fusobacterium* a more likely diagnosis because it takes about one week before it can be detected [4]. Furthermore, because *F. necrophorum *has been detected using molecular methods in culture-negative cases, it is possible that the *Fusobacterium* species is largely responsible for LS even when not detected by culture and a negative culture does not exclude necrobacillosis [1]. PCR may be the best laboratory test as it is able to identify *F. necrophorum* in culture-negative patients; however, there are no commercially available *F. necrophorum*-specific PCR kits currently [1].

In our 10-year systematic literature review of LS cases with ophthalmological complications, nine cases (33.33%) had blood culture-confirmed *F. necrophorum*. This is presented in Table 3. Of these cases, four patients made complete recoveries [4, 21, 24, 35], while three patients had a remaining abducens nerve palsy upon discharge [12, 25, 26]. Two cases had unclear or unstated outcomes [18, 23]. Nine cases (33.33%) had Gram-positive cocci identified on blood culture and these cases tended to have poorer outcomes. This is outlined in Table 4. Seven of the nine cases had remaining ocular deficits or worse. One case, as outlined earlier, had MRSA bacteremia and did not regain function of the optic nerve so there was no return of the pupillary light reflex [17]. Two other cases were fatal with S. milleri bacteremia or methicillin-sensitive S. aureus (MSSA) bacteremia [19-20]. The remaining four cases of Gram-positive cocci bacteremia that had poor outcomes were discharged with a remaining gaze palsy, remaining diplopia, or remaining strabismus [9, 16, 22, 26]. Based on this systematic review of reported cases, it seems like LS with Gram-positive cocci bacteremia tended to have worse ophthalmic outcomes than cases with blood culture-confirmed *F. necrophorum*. Furthermore, while the literature and the definition outlined in this paper specifically discuss the importance of culturing *F. necrophorum* for LS diagnosis, it would seem that there was a higher proportion of cases with Gram-positive cocci when looking at this subset of patients with LS and the ophthalmological complications emphasized in this review. 

**Table 3:** Outcomes in Blood Culture-Confirmed F. Necrophorum Lemierre’s Syndrome (LS) Patients With Ophthalmological Complications From 2009 - 2019 F. necrophorum: Fusobacterium necrophorum

Outcomes In Blood Culture Confirmed F. Necrophorum LS Patients With Ophthalmological Complications From 2009 - 2019	
Total cases	9
Full recovery	4
Remaining abducens nerve palsy	3
Unclear/not stated	2

**Table 4:** Outcomes of Lemierre’s Syndrome (LS) Cases With Gram-Positive Cocci Bacteremia and Ophthalmological Complications From 2009 - 2019

Outcomes of LS Cases With Gram-Positive Cocci Bacteremia and Ophthalmological Complications From 2009 - 2019	
Total cases	9
Death	2
Permanent loss of pupillary light reflex	1
Remaining gaze limitation, diplopia, strabismus	4
Improvement with no remaining recorded deficit	1
Unclear/not stated	1

In terms of imaging for IJV thrombophlebitis and septic emboli, a combination of imaging modalities can be used. A chest x-ray will typically be nonspecific until cavitations are seen late in the disease, making it less useful in the context of LS [1]. MR venography has a sensitivity of 100% and specificity of 96%, so it is the most accurate tool for identifying all the septic emboli and the thrombophlebitis characteristic of LS; unfortunately, its cost makes MR venography an unrealistic screening tool [1]. The most convenient tool is ultrasound, which is readily available at the bedside but has low sensitivity relative to other imaging modalities like MRI or CECT. Thus, CECT is probably the best middle ground where it can readily find emboli and is easily available to clinicians [5]. Ultrasound can still play an important role in managing patients after LS is diagnosed by allowing clinicians to determine how effective the treatment regimen is. Overall, the best use of imaging modalities for patients with LS utilizes both CECT to diagnose and identify locations of thrombus formation and septic emboli and color Doppler to track the progression of the thrombus while the patient is being treated [1].

Treatment

Treatment of LS revolves around aggressive antibiotic treatment, anticoagulation therapy, and surgical management [7]. Without antibiotics, the natural course of LS, as described by Lemierre in his original 1936 paper, led to death within 12 days [2]. Subacute cases led to death within 40 - 60 days; both are no longer natural histories of LS since antibiotic therapy is readily available [1]. Delaying antibiotic treatment may increase mortality and will affect long-term morbidity in LS. It is important to note that LS can progress despite being treated with appropriate antibiotics that have good Gram-negative anaerobe coverage [4].

Studies have shown that all strains of *F. necrophorum* are sensitive to metronidazole, ticarcillin-clavulanate, cefoxitin, co-amoxiclav, and imipenem, while resistance to erythromycin is relatively common in 15% - 22% of cases [1]. *F. necrophorum* has intrinsic resistance to gentamycin, quinolones, and erythromycin with poor observed efficacy of tetracyclines [1]. Furthermore, clindamycin has weak bactericidal activity against *F. necrophorum* and may even only be bacteriostatic in vivo, especially when compared with metronidazole or imipenem [1]. This could be explained, however, by antibiotics having poor penetration into the clots that define LS, with studies showing that antibiotic levels in serum/tissue did not correlate with efficacy and that antibiotics that penetrated well into the fibrin clot were more effective [1].

There is no clinical data comparing the efficacy of various antibiotics in treating LS, but based on in vitro data and clinical evidence, carbapenems, beta-lactam/beta-lactamase inhibitor combinations, and metronidazole seem to be the most effective drugs [1]. Among these, metronidazole has good oral bioavailability, excellent coverage of all *Fusobacterium* species, and strong penetration into tissues, specifically, the CNS, making it appear to be the best choice [1]. Carbapenems or piperacillin/tazobactam with metronidazole has a 98% success rate with an average duration of four weeks of treatment [5]. If patients respond well during the first two to three weeks of intravenous (IV) antibiotics, they can then be switched to an oral treatment protocol (generally, metronidazole) for four more weeks once their clinical symptoms have settled [1, 5].

In our systematic review of the literature, we found that there were no cases where antibiotics were not used as a first-line treatment for patients that had LS with ophthalmological complications. Furthermore, there was only one case where two or more antibiotics were not used to treat the patient [38]. To better understand the antibiotic patterns used to treat patients with this atypical presentation of LS, we reviewed Table 1 and tallied the number of times each antimicrobial was used in separate cases. We then excluded any antimicrobials that had not been used in three or more individual cases and presented this data in Table 5. Metronidazole was the most commonly used antibiotic and was used in 11 separate cases (40.74%). Meropenem and ceftriaxone were each used in seven cases (25.93%), while vancomycin and clindamycin were each used in six cases (22.22%).

**Table 5:** Antibiotics Used to Treat Lemierre’s syndrome (LS) Cases With Ophthalmologic Complications From 2009 - 2019 (Only Includes Antimicrobials Used in Three or More Separate Cases)

Antibiotics Used to Treat LS Cases With Ophthalmologic Complications From 2009 - 2019 (Only Includes Antimicrobials Used in Three Or More Separate Cases)	
Metronidazole	11
Meropenem	7
Ceftriaxone	7
Vancomycin	6
Clindamycin	6
Amoxicillin-Clavulanate	4
Piperacillin-Tazobactam	3

Anticoagulation is the second arm of managing LS, although the benefits of anticoagulation therapy are unclear. The risks and benefits of anticoagulation therapy have not been adequately assessed in a controlled study and the incidence of the disease is too low, making it difficult to determine the true cost-benefit ratio of providing anticoagulation treatment [6]. In two reviews, LS patients who received anticoagulation did have favorable outcomes, and most patients receive anticoagulation treatment, usually low-molecular-weight heparin (LMWH), based on departmental protocols but there is no strong data to guide anticoagulation therapy [7]. It is speculated that thrombosis in more severe sites, like the cavernous sinus, requires more aggressive treatment strategies and this could be an indication where anticoagulation is recommended, even when there is no clinical data to support this decision in LS patients [5, 7]. In studies focusing on anticoagulation in sinus thrombosis, a 2002 meta-analysis of three small randomized clinical trials found a nonsignificant reduction in mortality [15]. A subsequent 2004 prospective study showed that heparin was used for patients with sinus thrombosis 80% of the time with 79% of patients recovering, 5% of patients experiencing major morbidity, and 8% of patients succumbing to mortality [15]. This study has since guided the treatment of sinus venous thrombosis and most neurologists tend to treat with anticoagulation when the diagnosis is confirmed [15].

In our systematic review, we tried to quantify the anticoagulation trends used to manage the treatment of LS patients with ophthalmological complications. This is outlined in Table 6. Seventeen patients (62.96%) received some form of anticoagulation treatment. While heparin was the most frequently used anticoagulant reported, only one case in the last five years used heparin when anticoagulation was specified [37]. This may reflect the trend away from heparin towards LMWHs and direct oral anticoagulants in the medical community.

**Table 6:** Anticoagulation Used to Treat Lemierre’s syndrome (LS) Patients With Ophthalmological Complications From 2009 - 2019

Anticoagulation Used to Treat LS Patients With Ophthalmological Complications From 2009 - 2019	
Total	17
Heparin	5
Low-molecular-weight heparin (enoxaparin, dalteparin)	3
Rivaroxaban	2
Warfarin	1
Unspecified	6

Surgical intervention is saved for extremely ill patients and is done to prevent further production of septic emboli [5]. Additionally, the defining septic emboli and abscess formation of LS cause poor penetration of antibiotics with many reports showing that the infection only begins to resolve after surgical drainage of the pus, regardless of location [1]. Thus, surgical intervention is a critical third pillar of management for the most severe cases of LS and the specific surgical subspecialty that should be contacted is largely dependent on the location of the septic emboli. In our review, 19 patients (70.37%) received some type of surgical intervention ranging from the treatment of periodontal disease and incision and drainage of abscesses to otolaryngology and oculoplastic procedures.

For the management of the ophthalmic complications of LS, an oculoplastic consultation may be warranted. Oculoplastic intervention can be done to help treat the cranial nerve palsies that cause the most common ocular symptoms associated with LS (88.89% according to our data) (Table 2). For isolated palsies of CN III, IV, and VI, nonsurgical approaches using patches, prisms, and botulinum toxin injections can be used to acutely treat horizontal misalignment in the first six months (acute phase), after which surgery is considered for chronic cranial nerve palsies (defined as beyond six months) with the goals of gaining adequate realignment for binocular function, correcting ptosis, and correcting head position [39-40]. Among non-surgical treatment options, botulinum toxin, in particular, has been shown to cause a significant improvement in outcomes and is a minimally invasive technique that can be efficacious in the acute treatment of abducens nerve palsies [41]. A significant number of patients can have a full recovery with botulinum toxin alone and no surgery would be needed. If medical management alone fails over the first six months, then surgery would have to be considered and surgery in conjunction with botulinum toxin has superior outcomes relative to conventional management with surgery alone [42-44].

The abducens nerve is particularly vulnerable to intracranial pathologies, such as edema or inflammation, due to its long intracranial pathway. This makes it susceptible to the septic emboli and metastatic spread that defines LS, and as a result, it was the most frequently affected cranial nerve causing ophthalmic complications in our review (44.44% of cases) (Table 2). Because it is particularly vulnerable to many intracranial pathologies that can be more sinister than the acute defect itself, the initial management is to identify and treat the underlying cause. Afterward, acute management in the first six months relies on interventions, such as patching, compensatory prisms, or botulinum toxin, to prevent diplopia and abnormal head posture during the acute stage of presentation [41]. In a six-year retrospective study, Fitzsimons et al. identified 55 patients with abducens nerve palsies who were treated with botulinum toxin injections to the ipsilateral antagonistic medial rectus muscle with or without surgery. Seventy-two percent of patients obtained a significant benefit from botulinum toxin injection at any stage of their management [44]. Thirty-seven percent of patients in this series fully recovered with only botulinum toxin injections, suggesting that it has an efficacious role in the nonsurgical management of abducens nerve paresis cases, especially when given in the first six months of onset [41, 44]. Taken together, this suggests that botulinum toxin injections improve outcomes when given to patients whose natural history would include a full recovery or as an adjunct to surgery in patients whose natural history is one where there is a complete loss of function and no recovery without intervention.

Like isolated palsies affecting the other cranial nerves controlling the extraocular muscles, if there is a failure of improvement after six months, the palsy is considered chronic and corrective surgery is indicated. The surgical intervention chosen is critical in determining the optimal response. One must consider the severity of the paresis, the duration/onset, and the amount of motility limitation. By appropriately identifying these factors, good outcomes can be achieved with minimal need for secondary procedures or interventions. Bagheri et al. performed a 10-year retrospective study on abducens nerve palsies and compared outcomes between the group that received recession and resection (R&R) muscle surgery without transposition (51.5%), the group that received surgery with transposition procedures (24.2%), and the group that received botulinum toxin injections (24.2%); 21% of cases required a second procedure [41]. The final postoperative mean ± SD ocular deviation (prism diopters) after the first procedure was 5.5 ± 16.0 in the R&R group, 6.0 ± 9.8 in the transposition group, and 15.0 ± 20.0 in the botulinum group, which were all significant improvements relative to the preoperative measurements (56.9 ± 24.3 in the R&R group; 50.3 ± 16.8 in the transposition group; 44.3 ± 10.5 in the botulinum group) [41]. Head posture and limitation of mobility also significantly improved after each type of intervention [41].

## Conclusions

*Fusobacterium necrophorum*-induced Lemierre’s syndrome was relatively common before antibiotics but became “the forgotten disease” until a recent gain in incidence over the last 30 years. This could potentially be related to the decreased use of antibiotics for patients presenting with a sore throat. It is urgent that clinicians remember that non-streptococcal sore throat can still be of bacterial origins. While recent changes in prescription patterns may play a role in *F. necrophorum*-driven LS, the incidence of this disease is too low to warrant changing current antibiotic guidelines for pharyngitis/tonsillitis. Due to its low incidence and a broad spectrum of symptoms, Lemierre’s syndrome does not have an obvious clinical diagnosis and can confuse the clinician treating the patient. Communication between hospitalists, ophthalmologists, otolaryngologists, neurologists, and radiologists is critical towards quickly diagnosing the syndrome so that prompt antibiotics, anticoagulation, and surgical intervention can occur. Moving forwards, the most crucial aspect of managing Lemierre’s syndrome in the modern medical community is for physicians to be aware of this rare but life-threatening condition in order to expedite early treatment so that long-term morbidity and mortality can be minimized.
